# Physicochemical and functional properties of *Cucurbita maxima* pumpkin pectin and commercial citrus and apple pectins: A comparative evaluation

**DOI:** 10.1371/journal.pone.0204261

**Published:** 2018-09-20

**Authors:** Anna A. Torkova, Ksenia V. Lisitskaya, Ivan S. Filimonov, Olga A. Glazunova, Galina S. Kachalova, Vladimir N. Golubev, Tatyana V. Fedorova

**Affiliations:** 1 A.N. Bach Institute of Biochemistry, Research Center of Biotechnology of the Russian Academy of Sciences, Moscow, Russia; 2 Federal State-Owned Unitary Enterprise «All-Russian Research Institute for Optical and Physical Measurements», Moscow, Russia; 3 Russian National Research Center “Kurchatov Institute”, Moscow, Russia; 4 Science and Technological Park of the University of Girona, Girona, Spain; Fred Hutchinson Cancer Research Center, UNITED STATES

## Abstract

The physicochemical characteristics and functional properties of pumpkin (*Cucurbita maxima* D. var. Cabello de Ángel) pectin obtained by cavitation facilitated extraction from pumpkin pulp have been evaluated and compared with commercial citrus and apple pectins. *C*. *maxima* pectin had an Mw value of 90 kDa and a high degree (72%) of esterification.

The cytoprotective and antioxidant effects of citrus, apple and pumpkin pectin samples with different concentrations were studied *in vitro* in cell lines HT-29 (human colon adenocarcinoma) and MDCK1 (canine kidney epithelium). All pectin samples exhibited cytoprotective effect in HT-29 and MDCK1 cells after incubation with toxic concentrations of cadmium and mercury for 4 h. Pumpkin pectin increased the proliferation of cadmium-treated MDCK1 cells by 210%. The studied pectins also inhibited oxidative stress induced by 2,2′-azobis(2-methylpropionamidine) dihydrochloride (AAPH) in cell cultures, as determined by measuring the production of intracellular reactive species using dihydrochlorofluorescein diacetate (DCFH-DA). Pectin from pumpkin pomace had the highest (p < 0.05) protective effect against reactive oxygen species generation in MDCK1 cells induced by AAPH. Distinctive features of pumpkin pectin were highly branched RG-I regions, the presence of RG-II regions and the highest galacturonic acid content among the studied samples of pectins. This correlates with a considerable protective effect of *C*. *maxima* pectin against oxidative stress and cytotoxicity induced by heavy metal ions. Thus, *C*. *maxima* pectin can be considered as a source of new functional foods of agricultural origin.

## Introduction

Dietary fiber, particularly pectin, is among the most important components of the human diet. A lack of dietary fibers in everyday meal could negatively affect human health by increasing the risk of serious diseases including cancer [1–3]. Pectin has a wide spectrum of functional properties in addition to favorable effects on the gastrointestinal tract [4]. Due to their emulsifying and gelation properties, pectins are widely used by the food industry as stabilizers and gelling agents for mass-produced food [5]. Pectin from different sources exhibit antioxidative [6–8], anti-hypertensive [9], cytoprotective [10], immunomodulatory [11], hypocholesterolemic, hypoglycemic, prebiotic and other activities [12,13], that provide a promising basis for a wide range of functional foods.

Pectin is known to exhibit antioxidant activity [6,14]. The antioxidant activity of the polysaccharides could be affected by their structural properties or the presence of noncarbohydrate components [8,15], including phenolic compounds [14]. In addition to its own antioxidant effect, one of functions of pectin is to transport dietary antioxidants (vitamin C, carotenoids, and phenolic compounds) in the gastrointestinal tract and protect them from degradation in the acidic environment of the stomach [16,17]. Thus, intestinal epithelial cells are key targets for the antioxidant action of pectic polysaccharides in the human body and are an optimal model for testing the antioxidant effects of pectin. Among the commercially available human cell lines, the most popular intestinal epithelium models are HT-29 (intestinal adenocarcinoma) and Caco-2 [18]. Additionally, due to its ability to form monolayers, the MDCK1 (Madin Darby canine kidney) cell line of nonintestinal origin is widely used as a model for the intestinal epithelium [19–21].

Pectins are a structurally heterogeneous group of soluble dietary fibers with a high galacturonic acid (GalA) content. The latter forms acidic and neutral polymers. The majority of pectins include acidic linear homogalacturonan polymer containing 100 to 200 residues of galacturonic acid connected via (1→4)–α-glycoside bonds, which is the simplest structural element of pectin. In addition, homogalacturonan side chains can be esterified by linear or branched rhamnose oligosaccharides (rhamnogalacturonans I), and regions of alternating D-galacturonic acid and L-rhamnose residues substituted with various sugars can be present in the pectin structure (rhamnogalacturonans II) [22].

The structural features of pectin largely determine its technological and biofunctional properties. Low-esterified pectin readily forms complexes with divalent metals, including ions of toxic elements (mercury, lead, and cadmium), which leads to a decrease in the cytotoxic effects of heavy metals [23,24]. The antioxidant activity of pectin is reportedly related to the phenolic and total sugar (especially glucose) content [25,26] in the homogalacturonan chains, and is also influenced by the degree of esterification [6]. The hypocholesterolemic effect depends on the viscosity of pectins, which correlates with the degree of esterification and influences the rate of glucose absorption. The anti-cough effect of pectin appears to be correlated with the degree of branching [27]. Pectins rich in GalA effectively chelate heavy metals and reduce the adhesion of pathogens [12,28].

The plant source and extraction method are important factors determining pectin properties. Variations in extraction methods and conditions can yield fractions with different molecular weight, degree of esterification and branching, and phenol and sugar contents [29]. The most widely used method, hot acidic extraction, mostly preserves the original pectin structure but also leads to environmental concerns due to acid usage [30]. Other methods, such as enzymatic extraction or microwave-assisted extraction, can result in shorter polysaccharide fragments with higher yields [31,32].

Pumpkin is a promising source of pectin with distinctive features such as high degrees of esterification and branching. Antidiabetic, immunomodulation, antitussive, and antioxidant activities have been reported for pectin fractions extracted from different species of pumpkin [33–35]. Variations in the extraction methods influence the physicochemical properties and bioactivity of pumpkin pectin [29,32,36].Therefore, new extraction methods enable the acquisition of pectins with different functional properties.

In this work, we focus on the characterization of pectin from *Cucurbita maxima* D. var. Cabello de Ángel pumpkin. The method of choice for pectin extraction was a novel cavitation facilitated method that provided good pectin yield without additional acid and enzymatic treatments. Properties of the obtained pumpkin pectin were compared to those of commercial apple and citrus pectins. The obtained results revealed the interdependence between the structural features and functional properties of the pectins.

## Materials and methods

### Materials

Ripe pumpkin *C*. *maxima* D. var. Cabello de Ángel (EU registration No. EC ESb31) vegetables were harvested from the Valencia region of Spain (august-september 2015). The seeds and placenta were removed, and the resulting pumpkin pulp with peel was washed by a tap water and cut into pieces of 1–2 cm^3^.

Specially prepared water with electrical volume resistivity not less than 10 MΩ/cm at 20°C was used as an extracting agent. pH of the extraction media was within the range of 3.7–4.2. The pectin extraction process was a result of cavitational activation of water molecules with formation of active H_3_O^+^ radicals [37].

Highly methoxylated (the degree of esterification >50%) commercial citrus pectin APC105 LV (CP) and apple pectins (APA103 and APA104) were purchased from Yantai Andre Pectin Co., Ltd. (China) for comparison.

### Chemicals

Cd(NO_3_)_2_×4H_2_O, Hg(OCOCH_3_)_2_, MTT, DMSO, and DCFH-DA, boric acid, silver nitrate, alizarin, cresol red, phenol red, bromthymol blue, 3,5-dimethylphenol, D(+)-galacturonic acid monohydrate, gallic acid monohydrate, sodium carbonate, Folin-Ciocalteu’s phenol reagent were procured from Sigma-Aldrich Company (St. Louis, USA); DMEM and EMEM culture medias, Hanks balanced salt solution (HBSS), L-glutamine, trypsin-EDTA, penicillin, streptomycin were obtained from PanEco, Ltd (Russia); fetal bovine calf serum was obtained from HyClone (USA). Glacial acetic acid, concentrated hydrochloric, nitric, phosphoric and sulfuric acids were purchased from Acros Organics (Belgium).

All the other chemicals used in the study were of the highest purity available commercially or analytical grade.

### Pectin extraction

Pectin extraction was performed according to [38] Freshly pressed *C*. *maxima* pulp was mixed with water in the proportion of 1:(8–12) (w/v). The pectin extraction process was performed at 65–70°C during 15–30 min using a rotor-pulsation device MT-1500 (Kinematica, Switzerland). This resulted in disintegration of solid phase and high rate pectin extraction into liquid phase.

Further processing consisted in multi-stage filtration to remove the solid phase, purification on kieselguhr filter, filtration on membrane filters in ultra- and diafiltration modes to remove low-molecular weight impurities Cleared and partly concentrated pectin-containing liquid phase was further subjected to low-temperature concentration. The resulting concentrate was spray dried to obtain high grade purity pectin powder. For details see S1 Fig. The comparison between purified pumpkin pectin and commercial citrus and apple pectins is presented at S2 Fig.

Commercial pectins were washed with 98% isoamyl alcohol.

### Physicochemical characterization of pumpkin

The diameter, total weight and the contents (w/w) of the pulp with peel, seed, and placenta were determined using average measurements of 15 ripe vegetables (Table 1). The protein (N×6.25), moisture, fat and total sugar contents of the pumpkin pulp with peel, the sum of hydropectin and protopectin content were determined by approved AOAC methods (1995) [39].

**Table 1:** Physicochemical properties and composition of ripe *Cucurbita maxima* D. pumpkin. Values are means±s.d.

Parameter	Value
Vegetable diameter, cm	29±5
Vegetable weight, kg	2.4±0.3
Pulp and peel content, %	69.3±1.5
Placenta content, %	23.9±0.7
Seed content, %	6.8±0.2
Moisture, %	89.61±0.08
Protein, %	0.71±0.03
Fat, %	0.22±0.02
Total sugars, %	7.42±0.05
Pectin, %	1.27±0.08

### Physicochemical characterization of pectin

The nitrogen (Method 920.152), ash, acid-insoluble ash (Method 923.03) content and loss on drying of pectin were determined by approved AOAC methods (1990) [40]. Total phenol content in pectin after saponification with 1 M sodium hydroxide solution was determined according to [41] with gallic acid as a standard and expressed in mg of gallic acid equivalents (GAE) per g. Caroteniod content was determined by UV-Vis spectroscopy according to [42]. Three replicates were run for each pectin sample.

The pH of the 0.3 and 1.2% (w/vol.) pectin solutions at 25°C and amount of titrated carboxylic groups were determined with a pH meter (Seven Easy Multi, Mettler-Toledo, Germany). Pectin solutions were titrated with 0.1 M hydrochloric acid and 0.1 M sodium hydroxide solutions up to pH 2.5 and 10.5, respectively. Based on the amount of titrated carboxylic groups (C), p*K*a values of pectins were calculated according to Eq 1:

pKa=2pH−lg(C) (1)

The degree of esterification (DE) was assessed by a direct titrimetric method. Approximately 0.5 g of pectin was weighted into filtering crucible (porosity 2). After addition of 20 mL of acidified 75% ethanol (50 ml of concentrated hydrochloric acid per 1.0 L of 75% ethanol) the crucible was connected to the filtration system. Pectin was washed with 20 mL portions of acidified ethanol up until the negative reaction of filtrate with 0.1% ethanolic alizarin solution was observed. The residue was washed with 70% ethanol up until the negative reaction of filtrate with 0.05 M silver nitrate solution in 1 M nitric acid was observed. The residue on the filter was quantitatively transferred into the 250 mL conical flask by pouring distilled water heated up to 40°C. Final volume of pectin solution was adjusted to 100 mL. After pectin was completely dissolved 6 drops of Hinton indicator (mixture of 0.4% aqueous solutions of bromthymol blue, cresol red and phenol red 1:1:3 vol./vol./vol.) were added to the pectin solution and it was titrated with 0.1 M sodium hydroxide solution up until pink coloring was stable for at least 30 s and the resulting NaOH volume was recorded as V_1_. Then 50 mL of 0.1 M sodium hydroxide solution were added, the mixture was incubated for 1 h at room temperature for pectin deesterification with subsequent addition of 50 mL of 0.1 M hydrochloric acid. The excess of hydrochloric acid was titrated with 0.1 M sodium hydroxide solution and the resulting volume was recorded as V_2_. The DE was calculated according to Eq 2:

$$DE=(V_{2}/(V_{1}+V_{2}))\times 100$$ (2)

All experiments were carried out in three replicates.

### Analysis of monosaccharide composition

The sample (10 mg) was hydrolyzed in 2 M trifluoroacetic acid (2 mL) at 100°C for 8 h. The hydrolysate was cooled to ambient temperature and dried by nitrogen in a water bath (70°C). Hydroxylamine hydrochloride (10 mg) and pyridine (0.5 mL) were then added. The mixture was incubated at 90°C for 30 min with shaking. After cooling, 0.5 mL of acetic anhydride was added to the mixture and shaken at 90°C for 30 min again. The derivatives were obtained after filtering through the organic filter membrane. Quantification was performed by gas chromatography (6890N, Agilent Technologies Co., USA) equipped with a hydrogen flame ionization detector. The column used was DB1701 (30 m × 0.25 mm, 0.25 μm) and nitrogen served as a carrier gas. The following temperature program was used: initial temperature of 170°C was maintained for 2 min, then the temperature was raised to 250°C with a rate of 10°C per min, and the final temperature of 250°C was maintained for 10 min.

The determination of polygalacturonic acid (poly-GalA) content was performed by the 3,5-dimethylphenol (DMP) method according to [43], using D-galacturonic acid as a standard. Acid hydrolysis of pectin was carried out in 7 mL tubes with screw cap. The reaction mixture contained 50 μL of pectin solution (1 mg·mL^-1^), 650 μL of deionized water, 3.0 mL of 0.1% sodium chloride solution in concentrated sulfuric acid (96% w/w). The blank mixture contained 50 μL of deionized water instead of pectin solution. The standard sample contained 400 μL of 25–150 μg·mL^-1^ D(+)-galacturonic acid solution, 300 μL of deionized water, 3.0 mL of 0.1% sodium chloride solution in concentrated sulfuric acid (96% w/w). After the reaction mixture preparation, each tube was vortexed for 15 s and kept on ice. Two replicates were run for each pectin sample or concentration of standard. The reaction tubes were tightly sealed with screw caps and incubated for 15 min in water bath at 80°C. After cooling to room temperature, aliquots (120 μL) of each reaction mixture were transferred to the wells of non-binding 96-well microplate (Greiner Bio-One, Germany) in 12 replicates. 50 μL of glacial acetic acid was added to half of the replicates. The plate was inserted into a multi-detection microplate reader Synergy 2 (BioTek, USA), shaken for 10 s at a speed of 400 rpm with subsequent reading of the absorbance at 450 nm (30 s after the addition of acetic acid). 50 μL of 0.2% (w/v) DMP solution were added simultaneously to 6 wells, the plate was shaken for 10 s at 400 rpm in the Synergy 2 reader with subsequent determination of the absorbance at 450 nm (30 s after the addition of DMP solution). The initial absorbance values were subtracted from those observed after the addition of DMP solution, and the poly-GalA content in pectin was calculated on a dry and ash-free basis.

### Molecular mass distribution

Molecular mass of pectin samples was determined using gel filtration and viscosimetric methods (see section Intrinsic viscosity). The gel filtration method allows determining the molecular mass as well as homogeneity of the pectin tested. The gel filtration was performed using Sefadex G-75, with 0.05 M NaCl as an eluent.

### IR-Fourier spectroscopy

Infrared spectra were obtained using IR-Fourier spectrometer Agilent Cary 660 (Agilent Technologies) with the accessories (ZnSe crystals with a 45° incidence angle) for the Attenuated Total Reflectance (ATR) analysis. ATR spectra were collected by using 128 scans at 2 cm^-1^ resolution in a range of 4000–650 cm^-1^. A portion of the sample (1 ± 0.01 g) was placed on the ZnSe crystal for measurement.

### Intrinsic viscosity

The intrinsic viscosity was determined by capillary viscosity experiments. The pectin samples were dissolved by stirring in deionized water for 2 h at room temperature. The dissolved pectin was treated with cation exchange resin (Amberlite 120 H^+^) to protonate pectin before viscosimetric analysis and then the stock sodium chloride solution was added to obtain the desired salt concentration. 5 mL of the sample solution was filtered through a 0.22 μm nylon membrane and loaded into Ubbelode capillary (capillary no. 0a, JD, 0.53 mm) with *k* constant of 0.005. Viscosity measurements were carried out in a water bath at 35.00 ± 0.02 ^o^C, with the initial pectin concentration of 0.001–0.005 g·mL^-1^, using a Schott AVS-360 automatic dilution viscometer system.

The evaluation of pectin molecular weight by intrinsic viscosity analysis was performed according to [44]. The intrinsic viscosity was determined by extrapolating the [*η*]/*C* ~ *C* curve and the (ln *η*)/*C* ~ C curve to zero and averaging the value of the intercept. The average viscosimetric molecular weight was calculated by applying the Mark–Houwink equation (Eq 3), where *η* is the intrinsic viscosity.

$$[\eta ]=KM^{\alpha },$$ (3)

### Dynamic viscosity

Dynamic viscosity of pectin solutions was determined in LVDV-II+Pro rotary viscosimeter (Brookfield Engineering Laboratories Inc., USA) equipped with 00, 18, 34 spindles and special beakers. The temperature of pectin solutions was maintained in a cryothermostat FT-211-25 (Laboratory Equipment and Instruments, Russia). 10 (spindles 18 and 34) or 16 (spindle 00) mL of pectin solutions were added to the beakers of the viscosimeter and left to equilibrate. Dynamic viscosity of pectin solutions was measured at 25, 40 and 60°C. The solutions were tested in triplicate at share rates up to 25 s^-1^. The solutions of 0.5, 1.0 and 1.5% pectin in deionized water were prepared by incubation overnight at 4°C with subsequent stirring for 2 h at room temperature until complete dissolving of pectin. For analysis of pH effects 1.5% pectin solutions were prepared in Britton-Robinson buffer (0.04 M acetic acid, 0.04 M phosphoric acid, 0.04 M boric acid) with pH values 2.0–6.0. The solutions were incubated overnight at 4°C with subsequent stirring for 2 h at room temperature and adjustment of pH to nominal values by addition of 0.1 M hydrochloric acid or sodium hydroxide when needed.

### Differential scanning calorimetry (DSC)

Thermodynamic parameters of 1% pectin solutions were determined by differential scanning microcalorimeter DASM-4 (Puschino, Russia) at temperature range of 10–120°C, heating rate 2°C per min, and excessive pressure of 2.5 bar. Milli-Q water was run as a blank. Excess heat capacity scale in each experiment was calibrated through the use of Joule-Lenz law. Prior to DSC experiments, appropriate amount of Milli-Q water at 23–25°C was added to 1 g of pectin and the resulting solution was stirred for 6 h. The values of transition enthalpy (*ΔH*_*t*_) were calculated by integration of the peak in the excess heat capacity vs. temperature curve. DSC experiments were run in 3 replicates for each pectin sample.

### SAXS measurements

The scattering data were recorded on the small-angle X-ray scattering beamline BM29 BioSAXS at the ESRF (Grenoble, France) using radiation wavelength of 0.099 nm. X-ray scattered intensity was measured using a 2D detector Pilatus 1M (Dectris, Switzerland) with active area of 16.9 cm x 17.9 cm, i.e. 981 by 1043 array with pixel size of 172 microns. The sample to detector distance was 2.87 m corresponding to the scattering vector S in the range of 0.033 to 4.9 nm^-1^ (s = (4π/λ)*sinθ where 2θ is the scattering angle). The sample was poured into 1.8 mm diameter quartz capillary with wall thickness of few tens of micron while maintaining constant temperature during the time of measurement. Several successive frames (usually 10 or 20) of 1 s each were recorded for both the sample and the corresponding buffer. Data collection and processing were performed in an automated manner using specialized beamline software BsxCuBE and Atsas package. Each frame was carefully inspected for any sample damage induced by X-rays before calculating the average intensity and the associated experimental error. Each scattering spectrum was corrected for the detector response and scaled to the transmitted intensity. The measured scattering from the buffer was subtracted from the corresponding pattern of sample. Two series of experiments were performed: the scattering data were collected at 277 K from 1% and 0.12% solutions of pumpkin (PP), citrus (CP) and two types of apple pectin (APA103, APA104) in water and Hank’s solution, respectively. Pure water was used as a standard sample for intensity calibration.

### Cell studies

Cell culture lines HT-29 (human colon adenocarcinoma) and MDCK1 (canine kidney epithelium) were obtained from Russian cell culture collection (St.-Petersburg, Russia). HT-29 cells were kept in EMEM (Eagle's Minimum Essential Medium, PanEco, Russia) medium supplemented with 10% FCS (fetal calf serum, Biosera, France), 2 mM L-glutamine and antibiotics (penicillin and streptomycin). MDCK1 cell line was grown in DMEM (Dulbecco's Modified Eagle Medium, PanEco, Russia) medium supplemented with 10% FCS, 2 mM L-glutamine and antibiotics (penicillin and streptomycin). Cells were grown in a humidified atmosphere containing 5% CO_2_ and 95% air at 37°C in cell culture flasks (Nunc, USA). Cells were subcultured every 2–4 days after reaching confluency.

Fresh 2 mg·mL^-1^ pectin stock solutions were prepared for each experiment by adding warm HBSS (Hank's Balanced Salt Solution, PanEco, Russia) to pectin samples. Stock solutions were sterilized using 0.22 μm membrane filters (Millipore, USA). The pectin was further diluted with sterile HBSS to achieve final concentrations of 0.25–0.5 mg·mL^-1^.

### Cytoprotective assay

Cells were seeded at 96-well plates (Nunc, USA) and cultured overnight at humified atmosphere. Then culture media was removed and 100 μL of HBSS containing 600 and 400 μM Cd(NO_3_)_2_ were added to MDCK1 and HT-29 cultured cells, respectively. 25 and 15 μM Hg(OCOCH_3_)_2_ in 100 μL of HBSS was added to MDCK1 and HT-29 cells, respectively. Cells were incubated with heavy metal ions for 5 h, then solutions were removed and replaced with 100 μL of HBSS containing two concentrations (0.25; 0.5 mg·mL^-1^) of pectin samples for 4 h. Growth inhibition effect was further assessed by MTT assay.

### MTT assay

0.5 mg·mL^-1^MTT (3-(4, 5-dimethylthiazolyl-2)-2,5-diphenyltetrazolium bromide) stock solution prepared in HBSS was added to the cells, and the plates were incubated at 37°C in the dark for 3 h. The solution was then removed and 100 μL of 10% sodium dodecyl sulphate (SDS) solution dissolved in 0.01% HCl were added. The optical density of each well was measured at 540 nm with reference wavelength of 690 nm using a multi-detection microplate reader Synergy 2 (BioTek, USA). All values were corrected against the corresponding blank sample. Proliferation in sample wells was then expressed as % to control.

### DCFH-DA assay

The antioxidant properties of pectins were analyzed on the HT-29 and MDCK1 cells under oxidative stress induced by the exogenously generated peroxyl radicals resulted from thermal decomposition of AAPH (2,2′-Azobis(2-methylpropionamidine) dihydrochloride). Intracellular reactive species production was determined using 2,7-dichlorodihydrofluorescein diacetate (DCFH-DA) as described in [45]. 100 μL cell suspension with density of 10^6^ cells per mL was seeded on black high-binding 96-well plates and cultivated overnight. Cell culture medium was removed and 100 μL of HBSS with different pectin concentrations (0.25, 0.5 mg·mL^-1^) were added and then incubated for 2 h. HBSS was used as control. After removing the solutions of samples and control, 100 μL of DCFH-DA solution (10 μM dissolved in HBSS) was added to each well and then incubated for 30 min. This solution was then replaced with 1 mM AAPH solution dissolved in HBSS to induce intracellular oxidation. Oxidation was monitored immediately after AAPH was added and then with 30 min intervals for 90 min at 37°C in a multi-detection microplate reader Synergy 2 (BioTek, USA) with the emission wavelength set at 528 nm and the excitation wavelength set at 485 nm. The fluorescence in the samples and control wells was calculated according to Eq 4:

$$Fluorescence=Fluorescence_{ti}-Fluorescence_{t0}$$ (4)

where *Fluorescence*_*ti*_ is fluorescence measured at time point 30, 60, and 90 min and *Fluorescence*_*t0*_ is initial fluorescence measured immediately after the addition of AAPH. Reactive oxygen species (ROS) level was then expressed in % as ratio of sample fluorescence to control fluorescence.

### Statistical analysis

All experiments were performed in three replicates, and obtained data were analyzed by one-way ANOVA followed by Tukey’s HSD (honestly significant difference) post-hoc tests (p < 0.05). Whenever appropriate, data are represented by the mean ± standard deviation (s.d.).

## Results and discussion

### Extraction of pumpkin pectin

In the current study, the novel cavitation facilitated method of pectin extraction was adopted [38]. The yield of *Cucurbita maxima* pectin (PP) extracted by this method was 10%. This is comparable with other reported methods of pectin extraction from pumpkins. The yields of pectin obtained by the hot acid extraction method from *C*. *maxima*, *Cucurbita moschata* and *Cucurbita pepo* var. Styriaca were reportedly 5% [31], 8–20% [10] and 5–7% [46], respectively. Microwave-assisted pectin extraction from *C*. *maxima* and *C*. *pepo* var. Styriaca resulted in 11% [31] and 3.1–7.4% [46] yields. The yield of enzymatically extracted pectin from *Cucurbita mixta* was 10% [32]. Comparable yield of pectin together with environmental safety and absence of additional enzymatic treatment make our method very attractive for industrial application.

### Physicochemical characteristics of pectins

The functionality of pectin is closely related to its physicochemical characteristics, including poly-GalA content, degree of esterification (DE), molecular weight, etc. Physicochemical characteristics of the investigated pectins are summarized in Table 2. All four pectins in the study were highly methoxylated (DE over 50%) and had low nitrogen content (< 0.3%). The DE of the PP, commercial citrus pectin (CP), and commercial apple pectins APA104 and APA103 were 71.9%, 58.9%, 64.0% and 70.6%, respectively. The degree of methoxylation of the PP was higher than that reported for the *C*. *maxima* var. Volzhskaya Grey pectin obtained by enzymatic extraction (DE 53–65%) [30], different *C*. *mixta* pectin fractions obtained by enzymatic (DE 47.3%) and microwave-assisted (DE 55–63%) extractions [31,32], and *C*. *pepo* var Styriaca pectin obtained by hot acid extraction (DE 34–48%) [29].

**Table 2:** Physicochemical characteristics of the investigated pectin from *C*. *maxima* pumpkin and commercial citrus and apple pectins. Values are means ± s.d.

Characteristics		Pectin sample^a^			
		PP	CP	APA104	APA103
Weight loss from drying (105°C, 2 h), %		8.83±0.03	5.26±0.01	5.63±0.05	4.47±0.01
Ash, %^b^		2.79±0.05	1.52±0.04	1.40±0.04	1.38±0.01
Acid-insoluble ash, %^b^		0.80±0.03	0.63±0.01	0.49±0.02	0.50±0.02
Nitrogen, %^b^		0.26±0.01	0.24±0.01	0.25±0.01	0.26±0.02
Poly-GalA, %^c^		75.1±4.5	54.2±3.6	58.1±4.3	70.5±5.0
Total phenols, mg GAE/g		1.04±0.05	1.04±0.02	1.48±0.01	2.41±0.01
Degree of esterification (DE), %		71.9±0.7	58.9±0.4	64.0±0.5	70.6±0.3
pH (25°C)	0.3% solution	3.7	3.8	3.7	3.6
	1.2% solution	3.5	3.3	3.2	3.4
p*K*a (25°C)		4.77±0.13	4.73±0.30	4.50±0.29	4.50±0.13
Dynamic viscosity, 1% solution (cPs)		37.9±1.8	7.±0.3	12.0±2.4	18.1±1.0
Intrinsic viscosity [η] (dL·g^-1^)		4.5–6.3	2.6–2.7	3.4–3.9	2.5
Average molecular mass (kDa)		26–96	40	40	80

^a^APA104, APA104 –apple pectins, CP–citrus pectin, PP–pumpkin pectin.

^b^–calculated on a dry weight basis

^c^–poly-GalA content was calculated on a dry and ash-free basis.

On the basis of total ash content, the PP obtained in the current study by cavitation facilitated extraction with subsequent membrane separation was comparable to that of pectin obtained by enzymatic extraction from *C*. *maxima* var. Volzhskaya Grey (2.9–3.0) [30]. The total ash content of PP was nearly twice that of the commercial apple and citrus pectins. This could be attributed to the differences in the technologies used for extraction and separation of pectin from the raw plant materials. Commercial pectins are usually produced according to the classic technology of hot acid hydrolysis with subsequent alcohol precipitation of pectin [47–49]. In contrast, PP was extracted from pumpkin pulp and peel under milder conditions and separated from the extract by an ultrafiltration process. Thus, the di- and trivalent cations are partly retained by the pectic acid during the extraction of PP resulting in its relatively higher ash content (Table 2).

The *C*. *maxima* pectin obtained in the current work and APA103 were characterized by poly-GalA content values of 71–75%. This value was approximately 1.3 times lower for APA104 and CP (Table 2). The same values of total phenolic content were determined for PP and CP, while the phenolic content of the apple pectins was on average 1.9 times higher (Table 2). The total phenolic content of PP was 2–9 times lower when compared with pectin extracted from the pulp of *C*. *pepo* var. Styriaca [25,29]. No caroteniods were found in all pectin samples.

**Fig 1:**
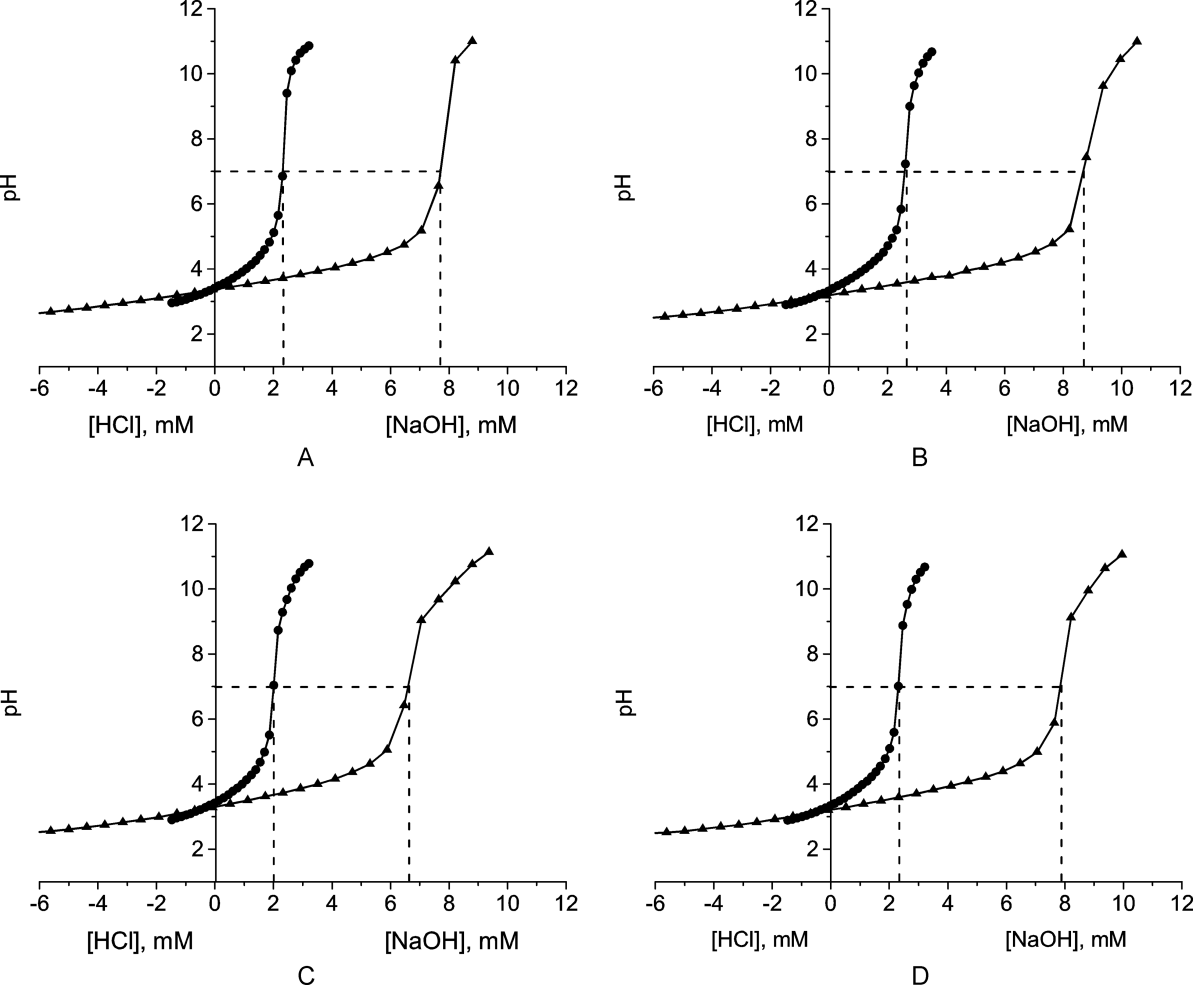
Acid-base titration curves of 0.3 (circle) and 1.2% (triangle) solutions of pectins from different sources. A–pumpkin pectin PP, B–citrus pectin CP, C–apple pectin APA103, D–apple pectin APA104.

The pH values of the pectin solutions were similar (Table 2). The data from acid-base titration (Fig 1) allowed the quantitative determination of titrated carboxylic groups and the p*K*a values (Table 2). The acid-base titration curves for the studied pectins (Fig 1) were characterized by a single point of inflection corresponding to the protonation-deprotonation of free carboxylic groups. The concentration of titrated carboxylic groups in 0.3 and 1.2% pectin solutions varied in the ranges of 1.6–2.2 and 6.2–8.2 mM, respectively. The p*K*a values of the pectins indicated they are weak acids. The content of the titrated carboxylic groups was in a good agreement with the content of poly-GalA and DE. The samples of CP and APA104 with lower DE values had higher amounts of the titrated carboxylic groups than the PP and APA103 samples. Conversely, APA103 had a lower poly-GalA content along with a higher DE value, and also had the lowest amount of titrated carboxylic groups (Fig 1D).

Molecular masses of the pectins were determined by gel filtration and viscosimetric methods. Under the conditions used, the intrinsic viscosity of commercial apple and citrus pectin solutions was lower than that of PP (Table 3), which was an indicator of a higher Mw of the PP, according to Kar and Arslan [50]. The molecular mass of PP obtained by gel filtration was in the range of 26–96 kDa, with prevailing higher molecular mass fractions. The obtained data were more consistent with the molecular weight of PP calculated using the random-coil model (Table 3). Parameters for the Mw calculation were taken from [51]. In the case of APA104 and CP, the differences in Mw calculated according to rod-like and random-coil models were insignificant. Their chromatographic masses were approximately 40 kDa. At the same time, the chromatographically obtained Mw of approximately 80 kDa for apple pectin APA103 was more consistent with the Mw calculated according to the random-coil model (Table 3).

**Table 3:** The molecular weight of PP, CP and the apple pectins as a function of intrinsic viscosity [η]. Values are means ± s.d.

Pectin sample*	Intrinsic viscosity [η] (dL/g)	Mw, kDa		
		Rod-like model (K = 0.00014, α = 1.34)	Random-coil model (K = 0.0234, α = 0.73)	Combined random-coil and rod-like model (K = 0.0955, α = 0.82)
APA103	3.9	64.2±6.0	87.8±8.2	139.8±14.7
APA104	2.5	46.3±5.3	48.1±5.4	81.9±8.8
CP	2.6	47.4±5.2	50.2±5.5	85.1±8.2
PP	4.5	72.2±7.7	108.6±10.5	169.1±16.0

*APA104 and APA104 –apple pectins, CP–citrus pectin, PP–pumpkin pectin.

### Structural characterization of the pectins

#### Monosaccharide composition of the pectins

Table 4 presents the data on monosaccharide composition of the pectins under investigation. Five different neutral sugars were found in all pectin samples: rhamnose, arabinose, galactose, glucose, and mannose. Xylose was also found in the apple pectins, and fucose was foundin the pumpkin pectin. The presence of fucose was observed by other researchers in the composition of pumpkin pectin, obtained by different extraction methods [29,36] and in the composition of apple pectin obtained using ultrasonic treatment [52].

**Table 4:** Monosaccharide composition of pectins.

Pectin samples*	Uronic acids (w/w, %)	Neutral sugar composition (w/w, %)							Degree of branching (Gal+Ara)/Rha
		Rha	Fuc	Ara	Xyl	Man	Glc	Gal	
PP	62.0	5.2	1.2	6.4	0.0	3.3	3.3	18.6	4.8
CP	67.7	6.3	0.0	1.4	0.0	4.8	1.9	17.9	3.1
APA103	62.9	6.6	0.0	4.2	6.2	5.3	5.4	9.4	2.1
APA104	52.8	9.5	0.0	2.5	7.8	14.1	5.7	7.6	1.1

*APA104 and APA104 –apple pectins, CP–citrus pectin, PP–pumpkin pectin.

Two types of polysaccharide regions were observed in the pectin samples: smooth region (homogalacturonan) and hairy region (rhamnogalacturonan). The most complex pectin structure, rhamnogalacturonan I (RG-I), has a backbone of alternating galacturonic acid and L-rhamnose residues, with branching structures consisting of D-galactose and L-arabinose chains attached to the L-rhamnose. The presence of rhamnose, galactose, and arabinose could indicate that all pectins contain RG-I regions with side chains, such as arabinan, galactan and arabinogalactan. However, PP has the highest (Gal+Ara)/Rha ratio, wich characterizes the degree of side chain branching of the pectin [53]. Fucose is probably an indicator of rhamnogalacturonan II (RG-II) side chains in PP [54]. The presence of glucose and xylose in the apple pectins could indicate the presence of xyloglucan as one of the side chains and the presence of xylogalacturonan [29,55,56]. The degradation of the RG-I region has an important impact on molecular mass. [52]. The reduction in the side chains of RG-I may be one of the reasons for the reduction in the Mw and the difference in molecular weight distribution. Indeed, among the studied pectin samples, the highest molecular mass and degree of branching were observed for PP.

#### FT-IR analysis

**Fig 2:**
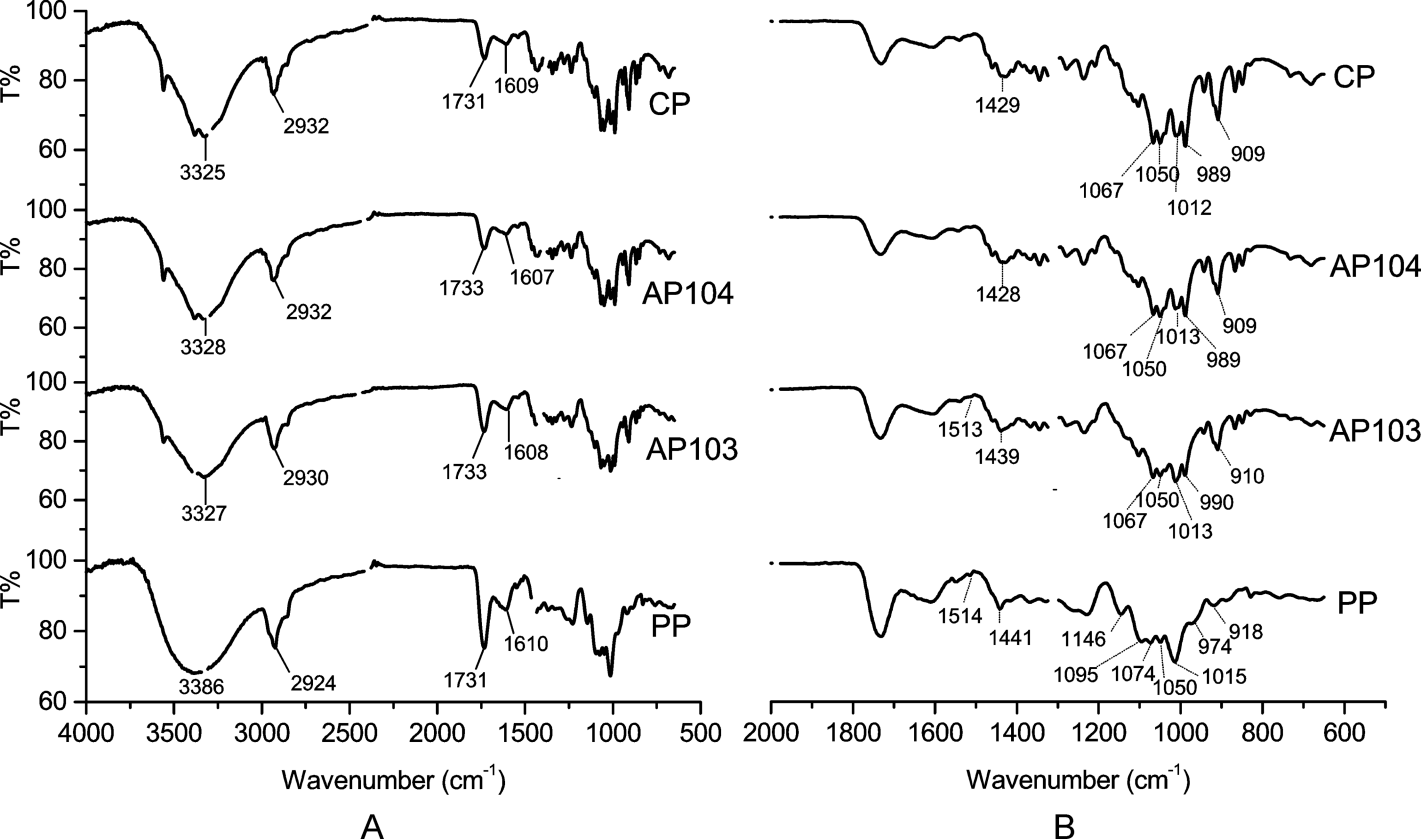
The FTIR-ATR spectra of the pumpkin, citrus and apple pectins. PP–*C*. *maxima* pectin; CP–citrus pectin; APA103 and APA104 –apple pectins. A–FTIR-ATR spectra in the 500–4000 cm^-1^ range; B–fingerprint region of pectins.

Analysis of the FT-IR spectra was performed to identify the major functional groups of the PP (Fig 2). The spectrum of PP was compared to those of commercial citrus and apple pectins (Fig 2).

The FT-IR spectrum of PP showed typical peaks for a number of specific groups. The intensive broad asymmetrical peak with a maximum at 3200–3600 cm^-1^ corresponds to valence oscillations of OH groups in the pectin molecule (Fig 2A). The area of aproximately 2926 cm^-1^ contains the peaks corresponding to oscillations of different groups containing C–H bonds. The area of 1500–2000 cm^-1^ corresponds to oscillations of C = O groups. The characteristic peaks of ester and carboxylic C = O vibrations were observed at 1730–1760 cm^-1^ (COO–R) and 1600–1630 cm^-1^ (COO–), respectively. It has been shown that the relative intensity of the last two peaks is related to the degree of methoxylation [8]. The relative intensity of the ester band (1731 cm^-1^ for PP and CP; 1733 cm^-1^ for the apple pectins) proportionally increased with the DE of pectin, whereas the intensity of the band corresponding to carboxylic stretching (1607, 1608, 1609 and 1610 cm^-1^ for APA103, APA104, CP and PP, respectively) decreased (Fig 2A). Comparative analysis of the PP spectrum with those of CP, APA103 and APA104 indicated that PP had the highest DE, which is in accordance with the data obtained by the direct titrimetric method (Table 2).

The region between 1200 and 800 cm^-1^ is referred to as the fingerprint region, and the intensity of the individual bands in this region are unique for each polysaccharide. According to Kostalova et al. [25], the fingerprint region of pumpkin pectin is represented by characteristic bands at 1145, 1103, 1077, 1050, and 1017 cm^-1^. At the same time, the absorption bands at 1145, 1104 and 1020 cm^−1^ are typical for pectin polymers, and the bands at 1077 and 1050 cm^−1^ correspond to vibrations of neutral arabinose and galactose-based glycans [57]. In that spectral region, the characteristic peaks (1146, 1095, 1074, 1050 and 1015 cm^−1^) were present in the PP spectrum (Fig 2B). Typical bands of arabinogalactans at 1146, 1074 and 1045 cm^−1^ [57] were the strongest in the PP spectrum in comparison with the apple and citrus pectin spectra (Fig 2B). These results are consistent with the neutral sugar composition of the studied pectins (Table 4).

#### SAXS data analysis

The scattering intensity from each pectin sample in a water solution was lower than that in Hank’s solution since the pectin concentration in water solution was also lower. In both types of pectin solutions, the excess of scattering intensity was observed at S-values below 0.3 nm^-1^, caused either by aggregates or by heterogeneities. The double-logarithmic plot of the scattering intensities vs. scattering vectors was analyzed to provide information about the fractal dimension of pectin scattering objects. The relationship between the scattering intensity and the scattering vector follows the local power law I~S^-D^, where D is the dimension of the mass fractal, and thus can be linearized when plotted on a double-logarithmic scale. The slope of the linear fit (D) was approximately -1.4 in the range of S from 0.3 to 2.6 nm^-1^ and -2 at S below 0.3 nm^-1^. Generally, the fractal dimension for a polymer is related to the Flory exponent (ν) as *D* = 1/ν so that the distance between the end points of the polymer chain (R), and the number of the monomers in the chain (N), are related as R~N^ν^. Although the fractal dimension is not a measure of overall size of scattering objects, it reflects the internal scaling of interatomic distances, inversely related to the excluded volume parameter and thus sensitive to favorable or unfavorable solvation of the polymer chain [58]. At scattering vector (S) values below 0.3 nm^-1^, only the PP sample in water solution preserved the same ν value of approximately 0.71 and thus the same properties of the polymer chain. It is interesting to note that the flexibility of the PP and APA103 polymer chains in Hank’s solution is slightly increased against water solutions since the corresponding ν values are decreased from 0.72 to 0.61 for PP, and from 0.72 to 0.64 for APA103. These two pectin samples are best described by the random coil model (Table 3) that is associated with higher flexibility of polymer [59]. Table 5 presents some structural characteristics of the pectin samples.

**Table 5:** Structural characteristics of the pectin samples.

Sample*	1% water solution				0.1% Henx solution			
	R_1c_, Å	R_2c_, Å	R_g_, Å	Mw, kD	R_1c_, Å	R_2c_, Å	R_g_, Å	Mw, kD
CP	29.7	9.9	67.0	116.0	36.2	12.6	68.6	157.0
APA103	19.1	9.3	66.2	99.0	28.7	11.3	63.3	129.0
APA104	29.5	9.4	66.7	112.0	10.9	7.6	56.4	73.4
PP	16.2	8.3	65.2	91.0	26.3	11.9	63.0	147

*APA104, APA104 –apple pectins, CP–citrus pectin, PP–pumpkin pectin.

The R_g_ value was derived from the size distribution function p(r) calculated from one-dimensional scattering curves of the pectin samples using an indirect transform program GNOM for small-angle scattering data processing [60]. The values of molecular weights of the studied pectin samples were estimated using the calculated V_porod_ values. Multiplicity of junction zones was most prominent when the Guinier approximation for the analysis of the cross-section was adopted. The R_c_ values allowed the estimation of the average size of junction boxes using the model of a rigid cylinder with a radius less than its length. Despite the overall comparability of the parameters determined in such way, they can nevertheless provide useful information about the sizes of bundles of chains. Along with the main peak corresponding to R_g_,the size distribution function of the pectin samples had another peak at 2 nm, which did not coincide with the calculated R_c_. Therefore, the broken-rod-like model, allowing polydispersity in the cross section of the junction zones, was applied. Two cross-sectional radii of gyration R_1c_ and R_2c_ of the scattering curves were evaluated in two Guinier ranges (0.08 < S^2^ < 0.3 and 0.43 < S^2^ < 1.2). According to the R_c_ values, the bundles of chains in all measured pectin samples both in water and in Hank’s solution were considerably larger than the diameter of a single sugar ring. There was a notable correlation between the size of junction zone and the values of mean molecular weights, i.e., the larger bundles of chains were observed in the samples with higher Mw. It is also worthwhile to note that the size of the junction boxes for the pectin water solutions was inversely related with pectin DE magnitude: the DE values of 72% (PP), 71% (APA103), 64% (APA104), 59% (CP) corresponded to R_c_ values of 16.2 Å for PP; 19.1 Å –APA103; 29.5 Å –APA104; and 29.7 Å –CP, respectively (Table 2, 5). Meanwhile, the pectins with similar DE and GalA content, such as CP and APA104 or PP and APA103, had comparable calculated molecular weights and R_c_ values (Table 5). This is, however, not in agreement with the results of molecular weight calculations according to the Mark–Houwink equation (Table 2), where the pectin samples PP and APA103 had larger molecular weights and [η] that of CP and APA104. This discrepancy could be the result of the RG-I region structure. It was shown that the presence of flexible arabinan, galactan, and/or arabinogalactan side chains in the RG-I region of polysaccharide molecules led to the formation of a sphere-like compact macrostructure with a shorter hydrodynamic size [61]. This could also affect the Mw value calculated from SAXS data.

The R_c_ and corresponding Mw values of PP, CP and APA103 were increased in Hank’s solution but to different extents. In the case of APA104 these values decreased in Hank’s solution. Apparently, the salts in Hank’s solution hindered electrostatic repulsions caused by the negatively charged uronic acid side chains, and, therefore, reduced the space occupied by individual polymer chains [62]. APA104 chains became closely packed, which suggests that electrostatic interactions determine the distance between APA104 polymer chain aggregates. The same effect was shown for soluble polysaccharides of soy in salt-containing solutions [63]. The increase in molecular weight observed for the other pectin samples (PP, CP and APA103) could be attributed to the increased size of junction zones. The largest increase in R_c_ and corresponding Mw value was observed for PP. Since PP had the largest degree of branching among the studied pectin samples, it is safe to suggest that the growth of the junction zones in Hank’s solution of PP was caused by the lateral association of previously existing zones rather than their elongation. It was previously shown that SAXS data with a tendency of divergence in the low angle region (< 0.3 nm^-1^) are an indication of large aggregates with a broad size distribution [64]. Thus, it can be argued that electrostatic interactions are the primary cause of gel formation in the case of apple pectin APA104, whereas in the cases of PP, CP and APA103 gel formation appears to occur through hydrogen bond formation and hydrophobic interactions between various regions of the pectin molecules. This agrees with the thermodynamic parameters of the studied pectin samples: the strongest interactions between polysaccharide chains were observed for APA104 (see below).

### Thermodynamic and rheological properties of the pectins in aqueous solutions

**Fig 3:**
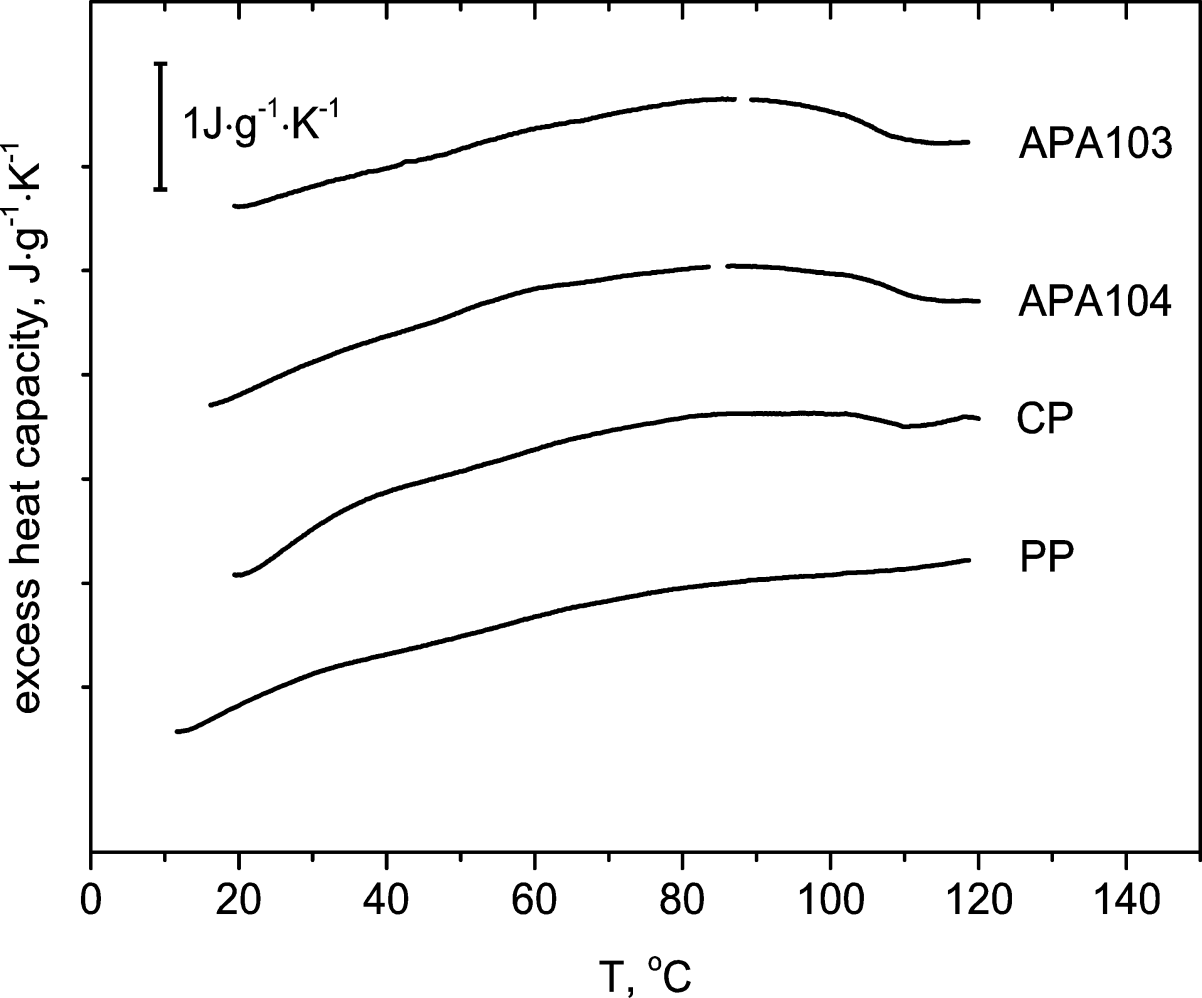
Excess heat capacity vs. temperature curves obtained by differential scanning calorimetry of 1% pectin solutions. 1—apple pectin APA103; 2—apple pectin APA104; 3—citrus pectin CP; 4—pumpkin pectin PP.

The thermodynamic properties of 1.0% aqueous pectin solutions were studied by DSC method. The DSC-thermograms obtained are given in Fig 3. No cooperative endothermic transitions were observed for the pectins studied. Instead, wide nonsymmetric peaks were observed in all DSC-thermograms (Fig 3). Such transitions are characteristic for polysaccharide systems with weak interactions between polysaccharide chains. Average transition enthalpy values were 4.7, 6.6, 7.5 and 8.5 J·g^-1^ for PP, CP, APA103 and APA104, respectively. Such low transition enthalpy values are typical for semihard domain polymers that are not able to make conformational rearrangements in the course of gel formation [65].

In 1% aqueous pectin solutions, formation of cross-links in the three-dimensional network was not accompanied by significant conformational entropy loss and, thus, a noncooperative type of gel formation was observed. No correlation between the values of transition enthalpy and the amount of titrated carboxylic groups in the pectins was observed. This indicates that the interactions between polysaccharide chains in 1% aqueous solutions of highly-methoxylated pectins are more likely determined not by the repulsion of the negatively charged carboxylates but by the distribution of the carboxylates along the pectin molecules and the prevalence of the linear (homogalacturonan) or branched-chain (primarily rhamnogalacturonans) polysaccharide regions in the pectin samples. Indeed, the relative degree of branching in the pectin molecules decreased in the series PP>CP>APA103>APA104 (Table 4). Thus, larger transition enthalpy values were characteristic for the pectins with lower degrees of branching. The gelation process is complexand involves electrostatic, hydrogen and hydrophobic interactions between the polysaccharide chains [66,67]. The gelation of highly methoxylated pectins takes place under conditions where electrostatic repulsions and water activity are reduced, in the presence of cosolutes at low pH. Most likely, gel formation in PP primarily involves hydrogen and hydrophobic bonds, which is consistent with the SAXS data.

**Fig 4:**
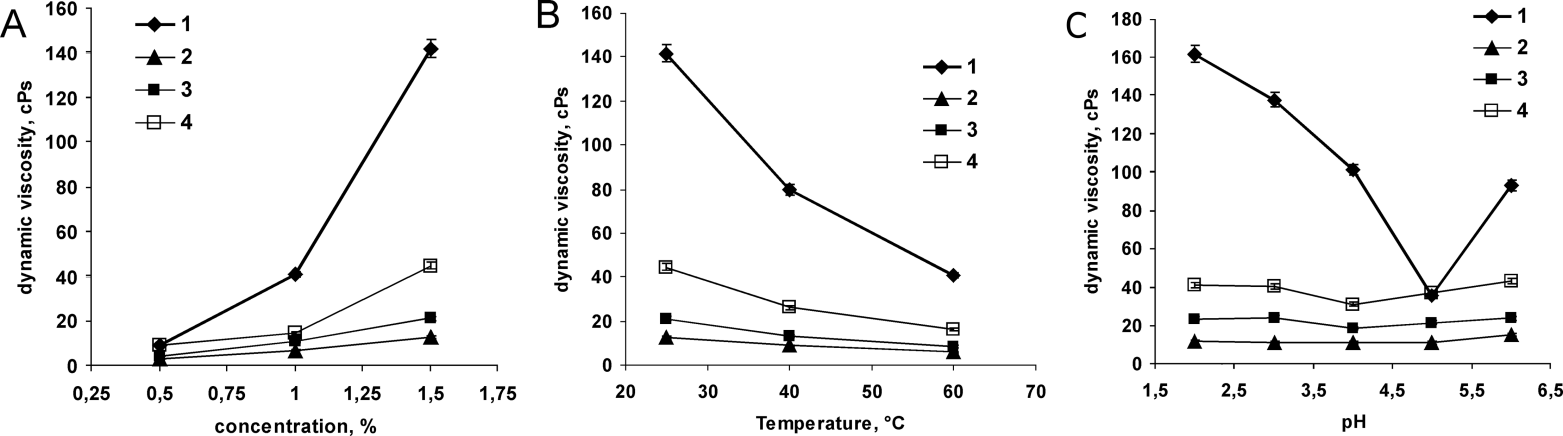
**Effects of concentration (A, 25°C), temperature (B, 1.5% solutions in water), and pH (C, 1.5% solutions in Britton-Robinson buffer, 25°C) on dynamic viscosity of the pectin solutions.** 1—pumpkin pectin PP, 2—citrus pectin CP, 3—apple pectin APA104, 4—apple pectin APA103.

Viscosity of aqueous pectin solutions is essential for their technological performance and beneficial biological effects [16,51]. Therefore, the impacts of temperature, concentration and pH on the viscosity of solutions were examined for PP and the three samples of commercial apple and citrus pectin (Fig 4). In concentration the range of 0.5–1.5%, the aqueous solutions containing APA103 and PP exhibited the highest dynamic viscosity among the pectins in the study (Fig 4A). This can be attributed to the higher molecular weight, poly-GalA content and DE of those pectins compared to APA104 and CP (Table 2). Additionally, PP, having the highest viscosity, was the sample with highest RG-I content and the only sample containing RG-II regions.

As it can be seen in Fig 4B, an increase in the temperature of the pectin solutions from 25 to 60°C led to notable decrease in their dynamic viscosity. Viscosity of the pectins with lower poly-GalA content and degree of esterification (CP and APA104) decreased only 2.1–2.5 times. The pectins with higher poly-GalA content and DE (PP and APA103) exhibited more prominent reductions in the dynamic viscosity values (2.7–3.5 times) upon increasing the solution temperature (Fig 4B). Moreover, PP was the most sensitive to temperature, having the highest RG-I content. A notable decrease in viscosity upon increasing the temperature is specific for the solutions of polymers with highly branched molecules. In addition, DE could also contribute to this effect by influencing the pectin structure and subsequently favoring the formation of hydrophobic interactions.

The effects of pH on the dynamic viscosity of the pectin solutions were examined in the range of 2.0–6.0 (Fig 4C). For all studied pectins, an increase of pH from 2.0 to 3.0–4.0 led to a decrease in dynamic viscosity values, while further increase in pH caused an increase in dynamic viscosity (Fig 4C). The lowest values of dynamic viscosity for the solutions of highly metoxylated pectins were observed at pH values close to their p*K*a (Fig 4C, Table 2). The apple pectins APA103 and APA104 with p*K*a approximately 4.5 exhibited the lowest viscosity values at pH 4.0 (Fig 4C). At the same time, the pumpkin and citrus pectins with higher p*K*a (4.7–4.8) exhibited the lowest viscosity values at pH 5.0 (Fig 4C). CP with the highest amount of titrated carboxylic groups among all of the studied pectins exhibited only a slight reduction (9.2%) of dynamic viscosity upon increasing pH from 2.0 to 5.0 (Fig 4C). The percentage of dynamic viscosity reduction for the apple pectins APA104 and APA103 upon increasing pH from 2.0 to 4.0 were nearly the same: 20.1 and 24.0%, respectively. The most profound decrease in dynamic viscosity (78.1%) was observed for the pumpkin pectin upon increasing of its pH from 2.0 to 5.0 (Fig 4C). The data obtained indicated that the dissociation of carboxylic groups caused rearrangements of three-dimensional pectic networks in solutions. The tendency toward remarkable reduction of dynamic viscosity of PP in solution at pH aproximately 5.0 provides additional opportunities for producing food and cosmetic products with tailored texture and viscosity.

### Cytoprotective properties of the pectins

#### Effect of the pectins on cadmium and mercury-induced cytotoxicity

**Fig 5:**
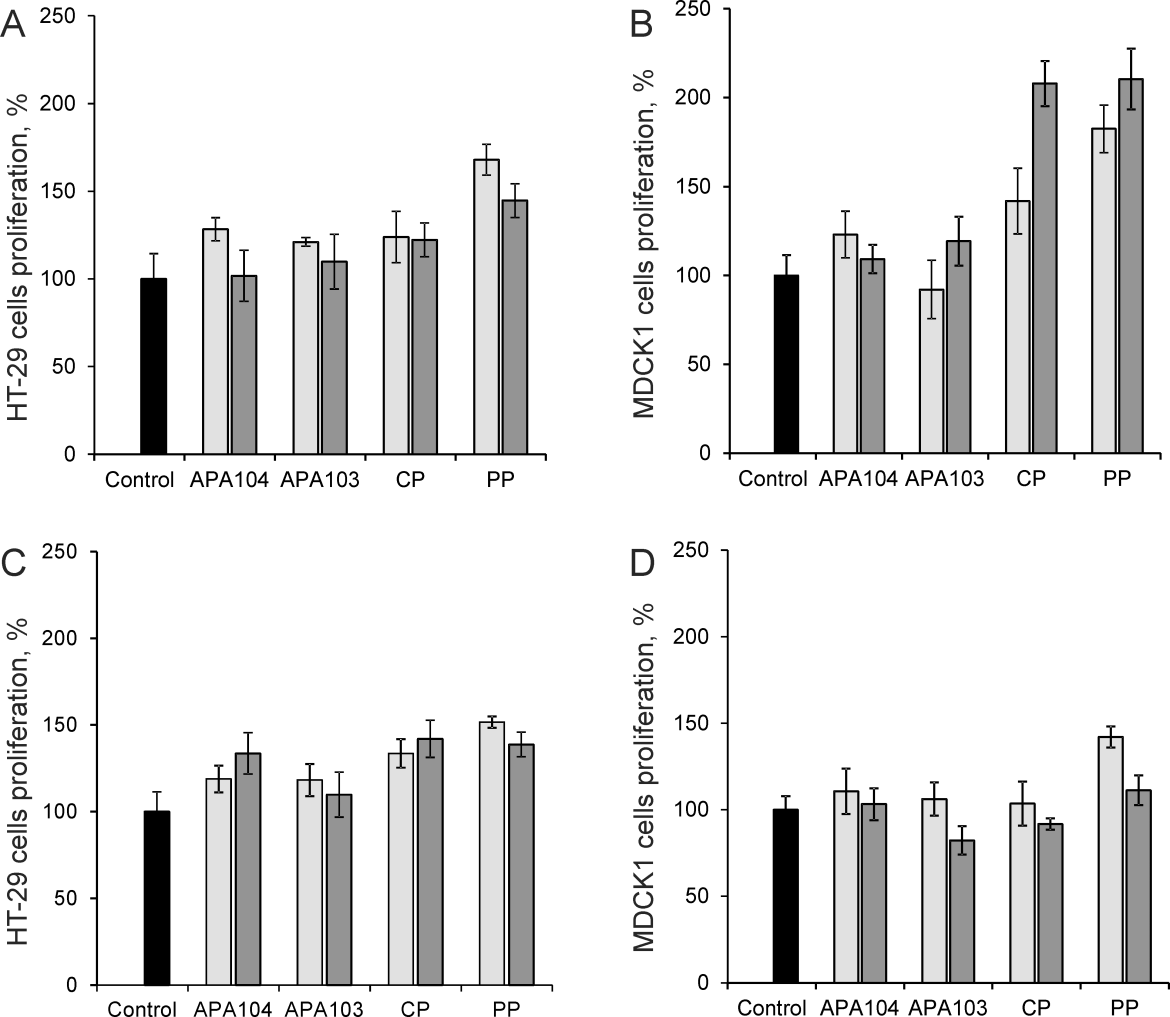
**Toxic effects of cadmium (A, B) and mercury (C, D) ions on HT-29 (A, C) and MDCK1 (B, D) cell cultures and protective effect of the pectin samples at two concentrations.** Light gray bars– 0.25 mg·ml^-1^ pectin concentration, dark gray bars– 0.5 mg·ml^-1^ pectin concentration.

Cadmium and mercury are widely distributed toxic metals in the environment. They possess cumulative toxicity and cause oxidative damage of tissues and DNA [68–70]. The cytoprotective effect against cadmium and mercury ions of all four studied pectins was assessed with two different cell lines: HT-29 and MDCK1, which are used as models of the intestinal epithelium [18–21]. Two different pectin concentrations were tested. The results are graphically presented on the Fig 5. The differences were considered statistically significant at p < 0.05.

In the case of cadmium ions (Fig 5A and 5B), the highest cytoprotective effect was observed for HT-29 cells for PP at both concentrations investigated (68% ± 9% at 0.25 mg·mL^-1^ and 45% ± 10% at 0.5 mg·mL^-1^). For MDCK1, the highest cytoprotective effect was observed for PP (111% ± 17%) and CP (108% ± 13%) at 0.5 mg·mL^-1^. At 0.25 mg·mL^-1^, PP outperformed (82% ± 13%) all of the other pectin samples.

In the case of mercury ions (Fig 5C and 5D), for both the HT-29 and MDCK1 cells, the highest cytoprotective effect was observed for PP at 0.25 mg·mL^-1^ (52% ± 7% and 42% ± 16%, respectively). However, the cytoprotective effect of PP against mercury ions was lower than that against cadmium ions for both concentrations investigated.

Although all of the studied pectins demonstrated reliable cytoprotective effect against cadmium and mercury ions, generally, the cytoprotective effects of the apple pectins were lower than those of CP and PP for both HT-29 and MDCK1 cells. The most pronounced cytoprotective effect was observed for PP, which that reduces the cytotoxicity of cadmium and mercury by at least 40% compared to the control.

The free carboxyl group concentration is considered the key factor controlling both the charge on pectin and its sorption capacity for heavy metals. The number of carboxyl groups in the studied pectins decreases in a series: CP > APA104 > PP > APA103 (Fig 1). However, the highest cytoprotective effect was observed for PP (Fig 5). Sorption properties of different pectins depend not only on the number of free carboxyl groups, but also on the viscosity, composition and structure of the side chains of pectin molecules [71]. Taken together, our results indicate that the sorption properties of pectin depend on its overall structure rather than individual parameters (i.e., there was no correlation between cytoprotective effects and DE for the studied pectin samples).

#### Effect of the pectins against AAPH-induced oxidative stress

**Fig 6:**
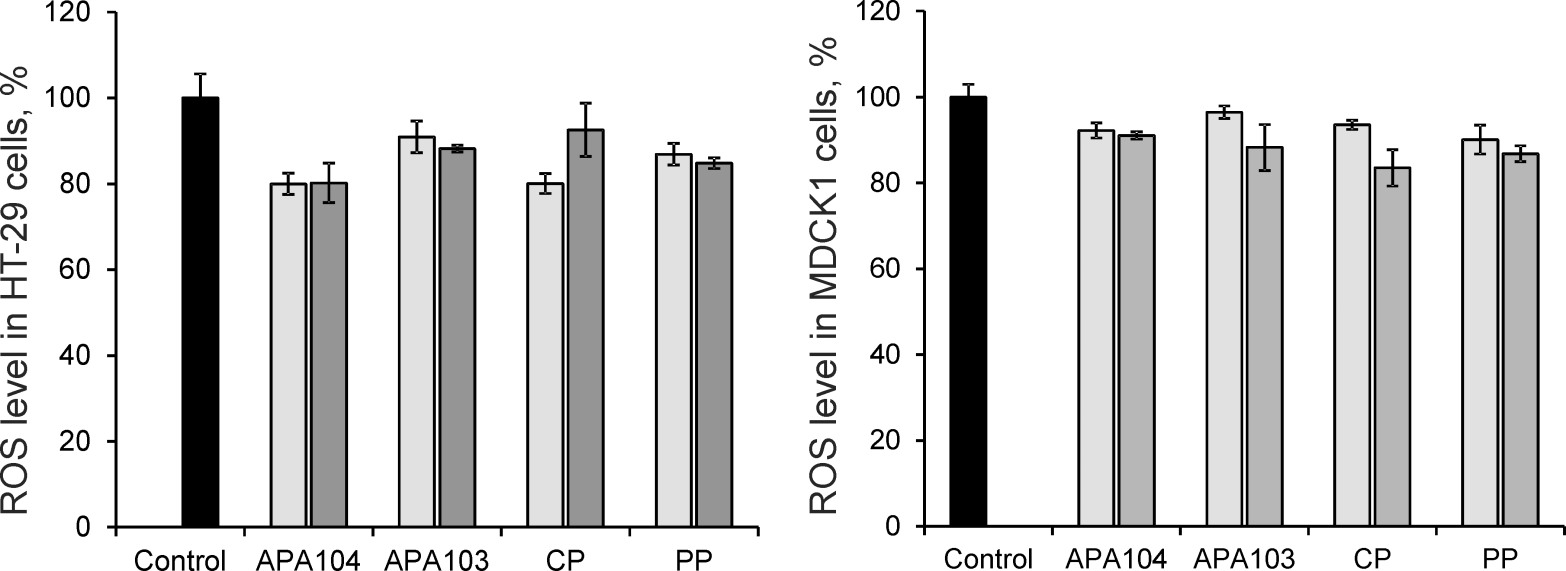
**Protective effects of the pectin samples on ROS generation in HT-29 (A) and MDCK1 (B) cells induced by AAPH**. Light gray bars– 0.25 mg·ml^-1^ pectin concentration, dark gray bars– 0.5 mg·ml^-1^ pectin concentration.

All pectin samples showed ROS-inhibiting activity in HT-29 and MDCK1 cells after preincubation of the cells for 90 min with the pectin samples at concentrations of 0.25 and 0.5 mg·mL^-1^. The differences were considered statistically significant at p < 0.05. In most cases, the antioxidant effect was concentration dependent (Fig 6). The antioxidant effect of the 0.5 mg·mL^-1^ pectin solutions in MDCK1 cells decreased in a series CP ~ PP > APA103 ~ APA104, which correlates well with the degrees of branching for these samples (Table 4). At a concentration of 0.25 mg·mL^-1^, PP exhibited the most pronounced decrease in the absolute value of DCFH-DA fluorescence intensity after 90 min of MDCK1 cells incubation with an azo-initiator.

Pumpkin pectin and apple pectin APA104 exhibited the most pronounced antioxidant effect on HT-29 cells, leading to a significant decrease in DCFH-DA fluorescence intensity in both studied concentrations (0.25 and 0.5 mg·mL^-1^).

Comparison of the results obtained in two cell models indicates the reliable antioxidant properties of PP on both cell lines. The differences in antioxidant effects of the pectins observed in both experimental models (tumor cells HT-29 and normal cultured cells MDCK1) apparently result from the differences in the structure and properties of the pectins from various plant sources. There are no significant correlations between antioxidant capacity *in vitro* and dynamic viscosity or degree of esterification. It was shown that phenols incorporated into pectin are essential for direct radical-scavenging effects of pectic substances [25,29]. Nevertheless, in our study, the higher total content of phenolic compounds did not result in the higher antioxidant capacity of pectin. Therefore, the antioxidant properties of the pectins appear to depend on the structure and composition of the side chains in RG-I regions. This is consistent with the results of other authors who reported that antioxidant properties of pectin depended not only on the phenolic content but also on other structural features. Thus, the prevalence of Gal residues in the side chains of the pectin macromolecules appeared to play an important role in the antioxidant activity [11,26,72]. Therefore, the differences in the cytoprotective effects of citrus, apple and pumpkin pectins in HT-29 and MDCK1 cells against oxidative stress and cytotoxicity induced by heavy metal ions resulted from different pectic oligosaccharide compositions, which depend on the extraction method, origin, molecular weight and degree of esterification. Similar results were obtained by Di et al. [28]. It was shown that the RG-I region of pectin containing arabinose-rich rhamnogalacturonic acids is responsible for *in vitro* prebiotic activity. At the same time, the maximal cytoprotective effect in HT-29 cells against Shiga toxin of *Escherichia coli* had pectin with a high GalA:Rha ratio in its composition.

## Conclusions

The present study demonstrates the efficiency of the cavitation-facilitated extraction method for the extraction of pectin from *C*. *maxima* pumpkin var. Cabello de Ángel, with the yield being 10%. Extraction conditions influenced the fine structure of pumpkin pectin. The present isolation protocols resulted in high molecular weight pectin, with a high degree of methoxylation. In this research, we showed that the poly-GalA content, different degrees of branching and esterification, and monosaccharide composition of the side chains altogether influence the rheological and functional properties of pectins.

In comparison with commercially available apple and citrus pectin samples, *C*. *maxima* pectin presented some individual features, such as like highly branched RG-I regions, the presence of RG-II regions and the highest poly-GalA content, along with a higher degree of methoxylation and a slightly higher viscosity and molecular weight. This correlates with pronounced antioxidant and cytoprotective properties of *C*. *maxima* pectin on cell models and indicates the potential of *C*. *maxima* pumpkin as a source of new functional foods with agricultural origin.

## Supporting information

S1 Fig Scheme of pectin extraction process. (TIF)

S2 Fig Comparison of pectin samples. PP1—pumpkin pectin before diafiltration. PP2—pumpkin pectin after diafiltration. CP—commercial citrus pectin, APA103 and APA104—commercial apple pectins.
(JPG)

## References

[1] Kushi LH, Doyle C, McCullough M, Rock CL, Demark-Wahnefried W, Bandera E V., et al American Cancer Society Guidelines on Nutrition and Physical Activity for Cancer Prevention: Reducing the Risk of Cancer With Healthy Food Choices and Physical Activity. CA Cancer J Clin. 2012;62: 30–67. 10.3322/caac.20140 Available 22237782

[2] Pietrzyk L, Torres A, Maciejewski R, Torres K. Obesity and obese-related chronic low-grade inflammation in promotion of colorectal cancer development. Asian Pacific J Cancer Prev. 2015;16: 4161–4168. 10.7314/APJCP.2015.16.10.4161 26028066

[3] Kaczmarczyk MM, Miller MJ, Freund GG. The health benefits of dietary fiber: Beyond the usual suspects of type 2 diabetes mellitus, cardiovascular disease and colon cancer. Metabolism. Elsevier Inc.; 2012;61: 1058–1066. 10.1016/j.metabol.2012.01.017 22401879 PMC3399949

[4] Brownlee IA. The physiological roles of dietary fibre. Food Hydrocoll. Elsevier; 2011;25: 238–250. 10.1016/J.FOODHYD.2009.11.013

[5] Funami T. Next target for food hydrocolloid studies: Texture design of foods using hydrocolloid technology. Food Hydrocoll. Elsevier; 2011;25: 1904–1914. 10.1016/J.FOODHYD.2011.03.010

[6] Nara K, Yamaguchi A, Maeda N, Koga H. Antioxidative Activity of Water Soluble Polysaccharide in Pumpkin Fruits (*Cucurbita maxima* Duchesne). Biosci Biotechnol Biochem. 2009;73: 1416–1418. 10.1271/bbb.80529 19502750

[7] Torralbo DF, Batista KA, Di-Medeiros MCB, Fernandes KF. Extraction and partial characterization of *Solanum lycocarpum* pectin. Food Hydrocoll. Elsevier; 2012;27: 378–383. 10.1016/J.FOODHYD.2011.10.012

[8] Košťálová Z, Hromádková Z, Ebringerová A. Isolation and characterization of pectic polysaccharides from the seeded fruit of oil pumpkin (*Cucurbita pepo* L. var. Styriaca). Ind Crops Prod. Elsevier; 2010;31: 370–377. 10.1016/J.INDCROP.2009.12.007

[9] Baluja Z, Kaur S. Antihypertensive aroperties of an apple peel—can apple a day keep a doctor away? Bull Pharm Med Sci. 2013;1: 9–16.

[10] Yang X, Zhao Y, Lv Y. Chemical composition and antioxidant activity of an acidic polysaccharide extracted from *Cucurbita moschata* Duchesne ex Poiret. J Agric Food Chem. American Chemical Society; 2007;55: 4684–4690. 10.1021/jf070241r 17511465

[11] Kratchanova M, Nikolova M, Pavlova E, Yanakieva I, Kussovski V. Composition and properties of biologically active pectic polysaccharides from leek (*Allium porrum*). J Sci Food Agric. 2010;90: 2046–2051. 10.1002/jsfa.4050 20572062

[12] Holck J, Hotchkiss AT, Meyer AS, Mikkelsen JD, Rastall RA. Production and Bioactivity of Pectic Oligosaccharides from Fruit and Vegetable Biomass Food Oligosaccharides. Wiley-Blackwell; 2014 pp. 76–87. 10.1002/9781118817360.ch5

[13] Wicker L, Kim Y, Kim M-J, Thirkield B, Lin Z, Jung J. Pectin as a bioactive polysaccharide–Extracting tailored function from less. Food Hydrocoll. Elsevier; 2014;42: 251–259. 10.1016/J.FOODHYD.2014.01.002

[14] Wang J, Hu S, Nie S, Yu Q, Xie M, Wang J, et al Reviews on Mechanisms of *In Vitro* Antioxidant Activity of Polysaccharides. Oxid Med Cell Longev. 2016;2016: 1–13. 10.1155/2016/5692852 26682009 PMC4670676

[15] Yang B, Zhao M, Shi J, Yang N, Jiang Y. Effect of ultrasonic treatment on the recovery and DPPH radical scavenging activity of polysaccharides from longan fruit pericarp. Food Chem. Elsevier; 2008;106: 685–690. 10.1016/J.FOODCHEM.2007.06.031

[16] Dikeman CL, Fahey GC. Viscosity as Related to Dietary Fiber: A Review. Crit Rev Food Sci Nutr. Taylor & Francis; 2007;46: 649–663. 10.1080/10408390500511862 17092830

[17] Saura-Calixto F. Dietary fiber as a carrier of dietary antioxidants: an essential physiological function. J Agric Food Chem. American Chemical Society; 2011;59: 43–49. 10.1021/jf1036596 21142013

[18] Cencič A, Langerholc T. Functional cell models of the gut and their applications in food microbiology—a review. Int J Food Microbiol. Elsevier; 2010;141: S4–S14. 10.1016/j.ijfoodmicro.2010.03.026 20444515 PMC7173225

[19] Astashkina A, Mann B, Grainger DW. A critical evaluation of in vitro cell culture models for high-throughput drug screening and toxicity. Pharmacol Ther. Elsevier Inc.; 2012;134: 82–106. 10.1016/j.pharmthera.2012.01.001 22252140

[20] Altınelataman C, Koroleva O, Fedorova T, Torkova A, Lisitskaya K, Tsentalovich M, et al An in vitro and in silico study on the antioxidant and cell culture-based study on the chemoprotective activities of fish muscle protein hydrolysates obtained from European seabass and gilthead seabream. Food Chem. Elsevier; 2019;271: 724–732. 10.1016/J.FOODCHEM.2018.08.004 30236737

[21] Lisitskaya K V., Nikolaev I V., Torkova a. a. Popov VO, Koroleva O V. Analysis of functional properties of biologically active substances using eukaryotic cell models (review). Appl Biochem Microbiol. 2012;48: 525–540. 10.1134/S0003683812060087 23330384

[22] Ridley BL, O’Neill MA, Mohnen D. Pectins: structure, biosynthesis, and oligogalacturonide-related signaling. Phytochemistry. Pergamon; 2001;57: 929–967. 10.1016/S0031-9422(01)00113-3 11423142

[23] Dalia ME-N. Effect of using pectin on lead toxicity. J Am Sci. 2010;6: 541–554.

[24] Kartel MT, Kupchik LA, Veisov BK. Evaluation of pectin binding of heavy metal ions in aqueous solutions. Chemosphere. Pergamon; 1999;38: 2591–2596. 10.1016/S0045-6535(98)00466-4 10204240

[25] Košťálová Z, Hromádková Z, Ebringerová A, Polovka M, Michaelsen TE, Paulsen BS. Polysaccharides from the Styrian oil-pumpkin with antioxidant and complement-fixing activity. Ind Crops Prod. Elsevier; 2013;41: 127–133. 10.1016/J.INDCROP.2012.04.029

[26] Rao RSP, Muralikrishna G. Water soluble feruloyl arabinoxylans from rice and ragi: Changes upon malting and their consequence on antioxidant activity. Phytochemistry. Pergamon; 2006;67: 91–99. 10.1016/j.phytochem.2005.09.036 16289622

[27] Nosáľová G, Prisenžňáková Ľ, Košťálová Z, Ebringerová A, Hromádková Z. Suppressive effect of pectic polysaccharides from Cucurbita pepo L. var. Styriaca on citric acid-induced cough reflex in guinea pigs. Fitoterapia. Elsevier; 2011;82: 357–364. 10.1016/j.fitote.2010.11.006 21062638

[28] Di R, Vakkalanka MS, Onumpai C, Chau HK, White A, Rastall RA, et al Pectic oligosaccharide structure-function relationships: Prebiotics, inhibitors of Escherichia coli O157:H7 adhesion and reduction of Shiga toxin cytotoxicity in HT29 cells. Food Chem. Elsevier; 2017;227: 245–254. 10.1016/j.foodchem.2017.01.100 28274429

[29] Košťálová Z, Hromádková Z, Ebringerová A. Structural diversity of pectins isolated from the Styrian oil-pumpkin (Cucurbita pepo var. styriaca) fruit. Carbohydr Polym. Elsevier; 2013;93: 163–171. 10.1016/j.carbpol.2012.05.017 23465915

[30] Ptichkina NM, Markina OA, Rumyantseva GN. Pectin extraction from pumpkin with the aid of microbial enzymes. Food Hydrocoll. Elsevier; 2008;22: 192–195. 10.1016/J.FOODHYD.2007.04.002

[31] Yoo S, Lee B, Lee H, Lee S, Bae IY, Lee HG, et al Structural characteristics of pumpkin pectin extracted by microwave heating. J Food Sci. 2012;77: C1169–C1173. 10.1111/j.1750-3841.2012.02960.x 23106191

[32] Cui SW, Chang YH. Emulsifying and structural properties of pectin enzymatically extracted from pumpkin. LWT—Food Sci Technol. Academic Press; 2014;58: 396–403. 10.1016/J.LWT.2014.04.012

[33] Quanhong LI, Caili F, Yukui R, Guanghui H, Tongyi C. Effects of protein-bound polysaccharide isolated from pumpkin on insulin in diabetic rats. Plant Foods Hum Nutr. 2005;60: 13–16. 10.1007/s11130-005-2536-x 15898354

[34] Felton LD, Prescott B, Kauffmann G, Ottinger B. Antigens of vegetable origin active in pneumococcus infections. J Bacteriol. 1955;69: 519–528. Available: http://www.ncbi.nlm.nih.gov/pmc/articles/PMC357578/ 14381369 10.1128/jb.69.5.519-528.1955 PMC357578

[35] Nagai M, Sato T, Watanabe H, Saito K, Kawata M, Enei H. Purification and characterization of an extracellular laccase from the edible mushroom Lentinula edodes, and decolorization of chemically different dyes. Appl Microbiol Biotechnol. 2003;60: 327–335. 10.1007/s00253-002-1109-2 12436315

[36] Košťálová Z, Hromádková Z, Ebringerová A. Chemical evaluation of seeded fruit biomass of oil pumpkin (*Cucurbita pepo* L. var. Styriaca). Chem Pap. 2009;63: 406–413.

[37] Golubev VN. Acoustic cavitation in food engineering. Proc 7th Int Conf On Ultrasound. 1996 p. 174.

[38] Golubev VN. Process for obtaining pectin. ES2216722B2, 2005.

[39] AOAC International. Official methods of analysis of AOAC International 16th ed Cunniff P, editor. Arlington, Va: AOAC International; 1995.

[40] AOAC Intlernational. Official methods of analysis of AOAC International 15th ed Helrich K, editor. Arlington, Virginia, USA: Association of Official Analytical Chemists, Inc; 1990.

[41] Singleton VL, Rossi JA. Colorimetry of total phenolics with phosphomolybdic-phosphotungstic acid reagents. Am J Enol Vitic. 1965;16: 144–158. Available: http://www.ajevonline.org/content/16/3/144.abstract

[42] Lichtenthaler HK, Buschmann C. Chlorophylls and Carotenoids: Measurement And Characterization by UV-VIS Spectroscopy. Handb Food Anal Chem. 2005;2–2: 171–178. 10.1002/0471709085.ch21

[43] Luzio GA. Determination of galacturonic acid content of pectin using a microtiter plate assay. Proc Fla State Hort Soc. 2004 pp. 416–421.

[44] Hourdet D, Muller G. Solution properties of pectin polysaccharides II. Conformation and molecular size of high galacturonic acid content isolated pectin chains. Carbohydr Polym. Elsevier; 1991;16: 113–135. 10.1016/0144-8617(91)90098-W

[45] Elisia I, Kitts DD. Anthocyanins inhibit peroxyl radical-induced apoptosis in Caco-2 cells. Mol Cell Biochem. 2008;312: 139–145. 10.1007/s11010-008-9729-1 18327700

[46] Košťálová Z, Aguedo M, Hromádková Z. Microwave-assisted extraction of pectin from unutilized pumpkin biomass. Chem Eng Process Process Intensif. Elsevier; 2016;102: 9–15. 10.1016/J.CEP.2015.12.009

[47] Chan SY, Choo WS, Young DJ, Loh XJ. Pectin as a rheology modifier: Origin, structure, commercial production and rheology. Carbohydr Polym. Elsevier Ltd.; 2017;161: 118–139. 10.1016/j.carbpol.2016.12.033 28189220

[48] Ciriminna R, Chavarría-hernández N, Inés A, Hernández R. Pectin: A new perspective from the biorefi nery standpoint. 2015; 10.1002/bbb

[49] Adetunji LR, Adekunle A, Orsat V, Raghavan V. Advances in the pectin production process using novel extraction techniques: A review. Food Hydrocoll. Elsevier; 2017;62: 239–250. 10.1016/J.FOODHYD.2016.08.015

[50] Kar F, Arslan N. Effect of temperature and concentration on viscosity of orange peel pectin solutions and intrinsic viscosity–molecular weight relationship. Carbohydr Polym. Elsevier; 1999;40: 277–284. 10.1016/S0144-8617(99)00062-4

[51] Masuelli MA. Viscometric study of pectin. Effect of temperature on the hydrodynamic properties. Int J Biol Macromol. Elsevier; 2011;48: 286–291. 10.1016/j.ijbiomac.2010.11.014 21134395

[52] Zhang L, Ye X, Ding T, Sun X, Xu Y, Liu D. Ultrasound effects on the degradation kinetics, structure and rheological properties of apple pectin. Ultrason Sonochem. Elsevier; 2013;20: 222–231. 10.1016/j.ultsonch.2012.07.021 22982008

[53] Yapo BM. Rhamnogalacturonan-I: A structurally puzzling and functionally versatile polysaccharide from plant cell walls and mucilages. Polym Rev. 2011;51: 391–413. 10.1080/15583724.2011.615962

[54] Maxwell EG, Belshaw NJ, Waldron KW, Morris VJ. Pectin–An emerging new bioactive food polysaccharide. Trends Food Sci Technol. Elsevier; 2012;24: 64–73. 10.1016/J.TIFS.2011.11.002

[55] Fissore EN, Ponce NM, Stortz CA, Rojas AM, Gerschenson LN. Characterisation of fiber obtained from pumpkin (*Cucumis moschata* duch.) mesocarp through enzymatic treatment. Food Sci Technol Int. SAGE Publications Ltd STM; 2007;13: 141–151. 10.1177/1082013207077914

[56] Vincken J-P, Schols HA, Oomen RJFJ, McCann MC, Ulvskov P, Voragen AGJ, et al If homogalacturonan were a side chain of rhamnogalacturonan I. Implications for cell wall architecture. Plant Physiol. 2003;132: 1781–1789. Available: http://www.plantphysiol.org/content/132/4/1781.abstract 12913136 10.1104/pp.103.022350 PMC1540329

[57] uráková M, Capek P, Sasinková V, Wellner N, Ebringerová A. FT-IR study of plant cell wall model compounds: pectic polysaccharides and hemicelluloses. Carbohydr Polym. Elsevier; 2000;43: 195–203. 10.1016/S0144-8617(00)00151-X

[58] Bhattacharjee SM, Giacometti A, Maritan A. Flory theory for polymers. Journal of Physics Condensed Matter. 2013 10.1088/0953-8984/25/50/503101 24222476

[59] Chou TD, Kokini JL. Rheological properties and conformation of tomato paste pectins, citrus and apple pectins. J Food Sci. 1987;52: 1658–1664. 10.1111/j.1365-2621.1987.tb05900.x

[60] Semenyuk A V, Svergun DI. GNOM—a program package for small-angle scattering data processing. J Appl Crystallogr. International Union of Crystallography; 1991;24: 537–540. Available: 10.1107/S002188989100081X

[61] Yapo BM. Rhamnogalacturonan-I: A Structurally Puzzling and Functionally Versatile Polysaccharide from Plant Cell Walls and Mucilages. Polym Rev. Taylor & Francis; 2011;51: 391–413. 10.1080/15583724.2011.615962

[62] Stokke BT, Draget KI, Smidsrød O, Yuguchi Y, Urakawa H, Kajiwara K. Small-Angle X-ray Scattering and Rheological Characterization of Alginate Gels. 1. Ca−Alginate Gels. Macromolecules. American Chemical Society; 2000;33: 1853–1863. 10.1021/ma991559q

[63] Chivero P, Gohtani S, Ikeda S, Nakamura A. The structure of soy soluble polysaccharide in aqueous solution. Food Hydrocoll. Elsevier; 2014;35: 279–286. 10.1016/J.FOODHYD.2013.06.006

[64] Yuguchi Y, Urakawa H, Kajiwara K. The effect of potassium salt on the structural characteristics of gellan gum gel. Food Hydrocoll. Elsevier; 2002;16: 191–195. 10.1016/S0268-005X(01)00082-0

[65] Doublier J-L, Nayouf M, Tecante A, Loisel C. Flow and viscoelastic properties of cereal starch/hydrocolloid pastes and gels Yuryev VP, Tomasik P, Ruck H, editors. Starch: From polysaccharides to granules, simple and mixture gels. New York: Nova Science Publishers, Inc New York; 2004.

[66] Ventura I, Bianco-Peled H. Small-angle X-ray scattering study on pectin–chitosan mixed solutions and thermoreversible gels. Carbohydr Polym. Elsevier; 2015;123: 122–129. 10.1016/j.carbpol.2015.01.025 25843842

[67] Hiorth M, Kjøniksen A-L, Knudsen KD, Sande SA, Nyström B. Structural and dynamical properties of aqueous mixtures of pectin and chitosan. Eur Polym J. Pergamon; 2005;41: 1718–1728. 10.1016/J.EURPOLYMJ.2005.02.028

[68] Skipper A, Sims JN, Yedjou CG, Tchounwou PB. Cadmium chloride induces DNA damage and apoptosis of human liver carcinoma cells via oxidative stress. Int J Environ Res Public Health. 2016;13: 1–10. 10.3390/ijerph13010088 26729151 PMC4730479

[69] Wiggers G a, Peçanha FM, Briones a M, Pérez-Girón J V, Miguel M, Vassallo D V, et al Low mercury concentrations cause oxidative stress and endothelial dysfunction in conductance and resistance arteries. Am J Physiol Heart Circ Physiol. 2008;295: H1033–H1043. 10.1152/ajpheart.00430.2008 18599595

[70] Rani A, Kumar A, Lal A, Pant M. Cellular mechanisms of cadmium-induced toxicity: a review. Int J Environ Health Res. Taylor & Francis; 2014;24: 378–399. 10.1080/09603123.2013.835032 24117228

[71] Dronnet VM, Renard CMGC, Axelos MAV, Thibault J-F. Characterisation and selectivity of divalent metal ions binding by citrus and sugar-beet pectins. Carbohydr Polym. Elsevier; 1996;30: 253–263. 10.1016/S0144-8617(96)00107-5

[72] Popov S V., Ovodova RG, Golovchenko V V., Khramova DS, Markov PA, Smirnov V V., et al Pectic polysaccharides of the fresh plum Prunus domestica L. isolated with a simulated gastric fluid and their anti-inflammatory and antioxidant activities. Food Chem. Elsevier; 2014;143: 106–113. 10.1016/J.FOODCHEM.2013.07.049 24054219

