# Supplementary figures and images for: Physicochemical and functional properties of Cucurbita maxima pumpkin pectin and commercial citrus and apple pectins: A comparative evaluation
Source: PLoS One. 2018 Sep 20;13(9):e0204261. doi: 10.1371/journal.pone.0204261 (PMC6147495; doi:10.1371/journal.pone.0204261)

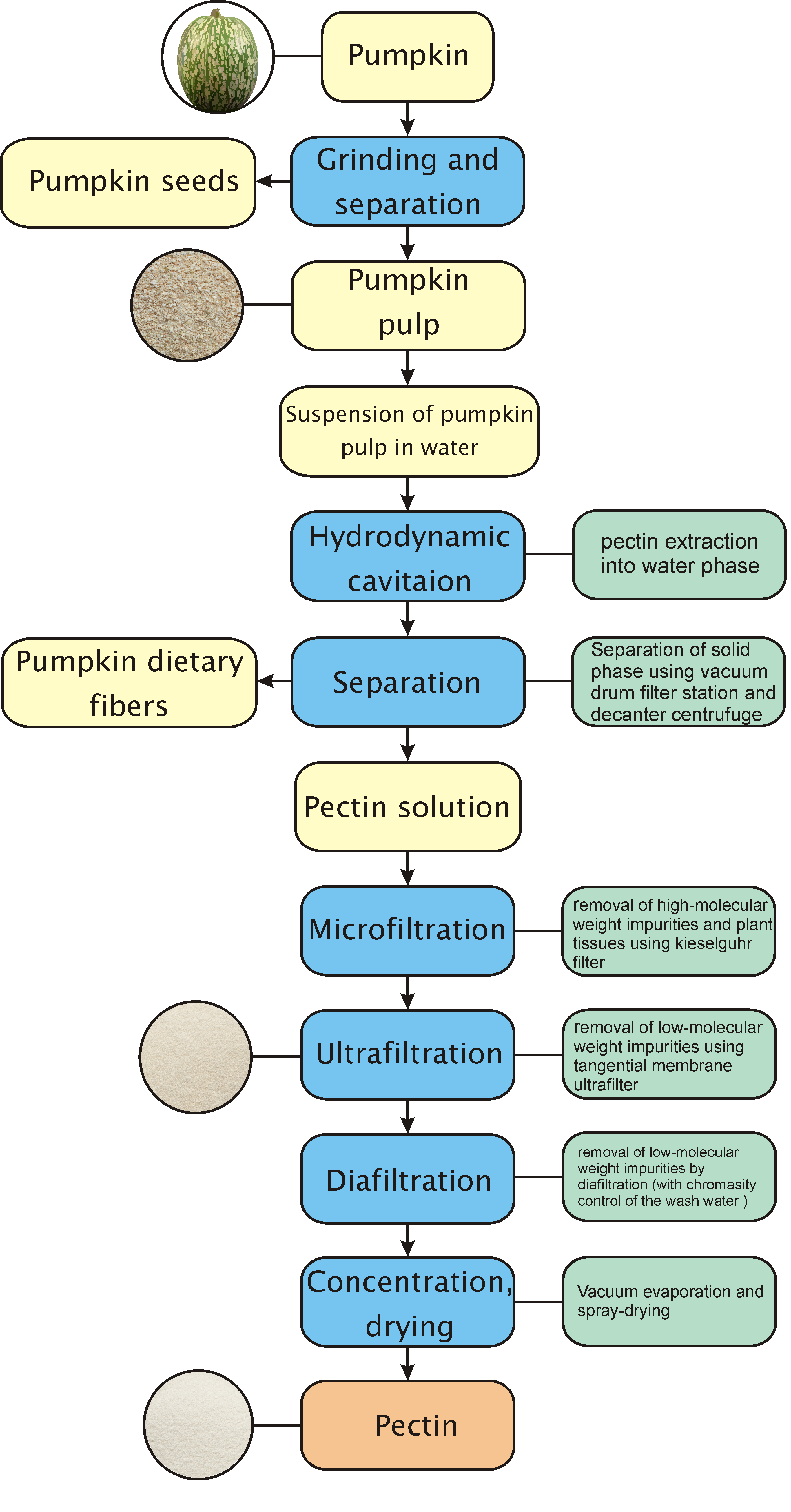

Supplement: S1 Fig — (TIF) [file pone.0204261.s001.tif]

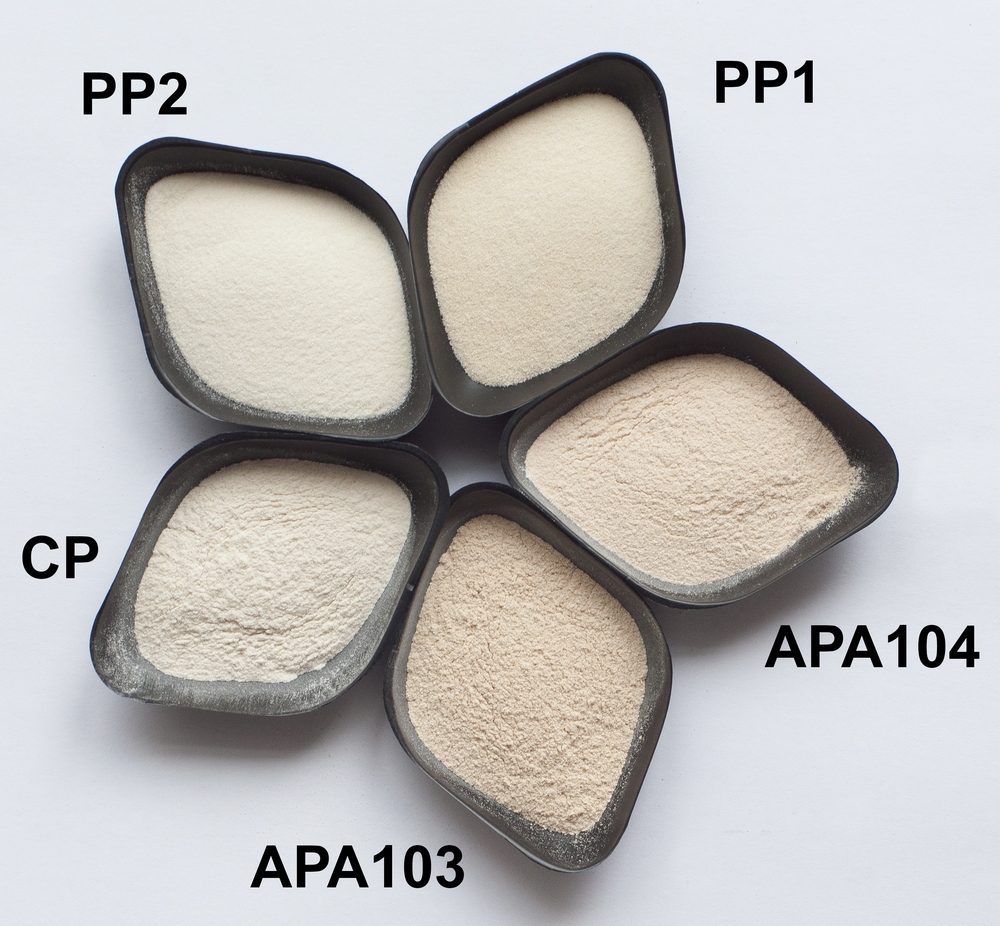

Supplement: S2 Fig — PP1—pumpkin pectin before diafiltration. PP2—pumpkin pectin after diafiltration. CP—commercial citrus pectin, APA103 and APA104—commercial apple pectins. (JPG) [file pone.0204261.s002.jpg]
